# Energy, nutrient and food content of snacks in French adults

**DOI:** 10.1186/s12937-018-0336-z

**Published:** 2018-02-27

**Authors:** Wendy Si Hassen, Katia Castetbon, Christine Tichit, Sandrine Péneau, Anouar Nechba, Pauline Ducrot, Aurélie Lampuré, France Bellisle, Serge Hercberg, Caroline Méjean

**Affiliations:** 1 Université Paris 13, Sorbonne Paris Cité, Centre de Recherche en Epidémiologie et Statistique, Equipe de Recherche en Epidémiologie Nutritionnelle, Inserm (U1153), Inra (U1125), Cnam, 74 rue Marcel Cachin, F-93017 Bobigny, France; 2 Université Libre de Bruxelles, Ecole de Santé publique, Centre de Recherche en Epidémiologie, Biostatistique et Recherche Clinique, Route de Lennik 808 – CP 598, 1070 Brussels, Belgium; 3 Institut National de la Recherche Agronomique (INRA) – UR 1303 Alimentation et Sciences Sociales ALISS, 65 boulevard de Brandebourg, F-94025 Ivry sur Seine, France; 4 Département de santé publique, Hôpital Avicenne, 125 rue de Stalingrad, F-93000 Bobigny, France; 5 MOISA, Université de Montpellier, INRA, CIRAD, CIHEAM-IAMM, Montpellier SupAgro, Montpellier, France

**Keywords:** Snack, Meal, Eating patterns, Eating occasions, Nutritional content

## Abstract

**Background:**

Snacking raises concern since it may lead to an additional energy intake and poor nutrient quality. A snacking occasion can be defined as any eating occasion apart from main meals, regardless of the amount or type of foods consumed. We described the frequency of snacking occasions according to daily timing in French adults, and compared them between each other, and with the main meals, in terms of energy intake, energy and nutrient density, and food content.

**Methods:**

This cross-sectional analysis included 104,265 adults from the NutriNet-Santé cohort. Food intake was estimated using 24-h records of weekdays. For each eating occasion, nutrient density and energy content and density were computed.

**Results:**

After weighting, 47.6% of our sample were men and mean age was 45.6 (15.3). Overall, 68% of participants ate at least one snack during the reported record, mainly in the morning or afternoon. Overall snack had a lower nutrient density [22.8 (SD = 278.3)] than main meals [25.8 (36.9) to 30.0 (30.4)]; but higher energy density [222.2 (163.3) kcal/100 g] than meals [133.9 (57.3) to 175.9 (99.6) kcal/100 g]. Morning snack was the snacking occasion with the lowest energy density [211 kcal/100 g], the lowest energy intake [104.1 kcal] and the highest nutrient density [60.1]. Afternoon and evening snacks had the highest energy loads [192.4 kcal and 207.6 kcal], but low nutrient scores [16 and 13, respectively]. The main food groups contributing to energy intake from snacks were fatty-sweet and sugary foods, fruit, hot beverages, and bread.

**Conclusions:**

Our findings highlight the frequency of snacking and the varying nutritional quality of snacks over the day. The morning snack was shown to be healthier than afternoon and evening snacks.

**Trial registration:**

This study was conducted according to guidelines laid down in the Declaration of Helsinki, and all procedures were approved by the Institutional Review Board of the French Institute for Health and Medical Research (IRB Inserm No. 0000388FWA00005831) and the French Data Protection Authority (Commission Nationale Informatique et Libertés No. 908450 and No. 909216). Electronic informed consent was obtained from all participants (Clinical Trial no. NCT03335644).

**Electronic supplementary material:**

The online version of this article (10.1186/s12937-018-0336-z) contains supplementary material, which is available to authorized users.

## Background

Snacking behavior, defined as any eating occasion apart from main meals, regardless of the amount or type of food consumed [1], is a common practice in Western countries [2–6]. Although the pattern of eating three main meals, characterized by structure and conviviality, continues to prevail in France [7–10], previous studies had shown that snacking is frequent among French adults and children [5, 11]. The effects of snacking and eating frequency on dietary quality, nutrient intake and health are ambiguous [1, 12]. In some studies, snacking or eating frequency were associated with better dietary quality and higher intake of vitamins, potassium or magnesium, but also carbohydrates [13–17]; others found inconsistent associations with intake of protein and fat [13, 16]. One Finnish study showed that when daily energy intake came mainly from snacking, daily dietary intake had lower nutrient quality, associated with higher intake of fructose and lower intake of micronutrients such as potassium and vitamin C [18]. Most studies have focused on the nutrient content of snacking. Previous works reported that the main foods consumed during snacking varied, i.e. desserts, sweet and fat products, juice and fruits, sweetened beverages, salty snacks, coffee, milk, nuts, etc. [3, 19, 20]. However, little is known about the contribution of foods to snacking in terms of energy and dietary quality [21, 22]. In a study conducted in the United States among working adults in 2010–2013 [21], energy intake from snacks came mainly from desserts and sweets, chips, crackers and fruits, while the major contributors to energy intake in Finnish adults in 2002 were sweet bakery goods, bread and milk products [18]. Such differences highlight the importance of social and cultural contexts in snacking practices. Differences in nutritional content may also vary according to the daily timing of snacking in the two countries. However, up until now, no study has explored together the nutrient content, energy density and food group intake associated with snacking according to time of day.

Snacking may represent a large percentage of the daily energy intake and may contribute to a positive energy balance [2, 19, 23] that could consequently lead to weight gain [24, 25]. The contribution of snacking to energy and food intake thus needs to be further elucidated. Our objectives were to describe the frequency of snacking occasions according to daily timing in French adults, and to compare them between each other, and with the main meals, in terms of energy intake, energy and nutrient density, and food content.

## Methods

### Sample and study design

Subjects were participants in the NutriNet-Santé study, a large web-based prospective observational cohort launched in France in May 2009. The study was implemented in the French general population targeting internet-using volunteers aged ≥18 years. The design, methods and rationale have been described previously [26]. Briefly, participants were included in the cohort once they had completed a baseline set of questionnaires assessing dietary intake, physical activity, socioeconomic and health status. As part of their follow-up, the participants completed the same set of questionnaires every year. This study was conducted according to guidelines laid down in the Declaration of Helsinki, and all procedures were approved by the Institutional Review Board of the French Institute for Health and Medical Research (IRB Inserm No. 0000388FWA00005831) and the French Data Protection Authority (Commission Nationale Informatique et Libertés No. 908450 and No. 909216). Electronic informed consent was obtained from all participants.

This cross-sectional study focused on 104,265 participants included in the NutriNet-Santé cohort study between May 2009 and January 2015, living in the French metropolitan area, who had completed at least two 24-h dietary records at baseline and with no missing data for the weighting procedure.

Socioeconomic (education, occupation, household income) and demographic (age, area of residence, marital status and presence of children in the household) data were collected at baseline using a web-based self-completed questionnaire, using categories consistent with the French National Institute of Statistics definitions and demographic variables [27–29]. The highest attained diploma defined the educational level [27]. The reported monthly household income was then divided by the number of household units (HU) [30].

### Assessment of dietary behaviors

At baseline, participants were invited to fill in 3 non-consecutive web-based 24-h dietary records, randomly assigned over a 2-week period (2 weekdays and 1 weekend day) [26, 31, 32]. The dietary record was completed via a validated interactive interface and designed for self-administration on the Internet [33]. The web-based dietary assessment method relies on an event-based approach, recording all foods and beverages (type and quantity) consumed at all eating occasions. On the web-based interface, only 4 initial categories of eating occasions are suggested and named as follows: breakfast, lunch, dinner, and “other eating occasion”. Participants were also asked to indicate time of each eating occasion. The participants estimated portion sizes for each reported food and beverage according to standard measurements (e.g., home containers, grams indicated on the package) or using validated photographs [34]. Values for energy, macronutrients and micronutrients were estimated using published nutrient databases and completed for recent market foods and recipes [35].

### Definition of meals and eating occasions

We categorized the eating occasions according to their nutritional content and self-reported time. Our first step was to associate all eating occasions with time periods. After noticing that almost all self-reported main meals (breakfast, lunch, and dinner) were occurring during specific time slots, we chose to define the following time periods: early morning slot (2 AM to 5 AM), breakfast slot (5 AM to 10 AM), late morning slot (10 AM to 11 AM), midday or lunch slot (11 AM to 2 PM), afternoon slot (2 PM to 6 PM), dinner slot (6 PM to 10 PM) and late night slot (10 PM to 2 AM).

Then, we analyzed the content of the self-reported main meals (breakfast, lunch and dinner): reported main meals which contain only a beverage or seasoning or only contain one food item which quantity consumed was lower than 20 g for breakfast or 50 g for lunch or dinner, were recoded as “other eating occasion”. When one or several other eating occasions and a main meal were reported in the same time slot, we compared the energy content of the other occasions to that of the main meal. The eating occasion with the highest energy value was considered as the main meal and the remaining ones were recoded as “other eating occasions”.

Finally, 8 categories of eating occasions were used: 3 main meals (breakfast, lunch, dinner) and 5 snacks (morning, midday, afternoon, evening and night snacks). Morning snacks include all other eating occasions than main meals occurring during the breakfast and late morning time slots. Midday snacks include all other eating occasions than main meal occurring during the midday slot. Afternoon snacks include all other eating occasions than main meals occurring during the afternoon time slot. Evening snacks include all eating occasions other than main meals occurring during the dinner time slot. Night snacks include all eating occasions other than main meals occurring during the late night and early morning time slots.

Daily overall snacking was defined by the occurrence of at least one snacking occasion during the 24 h record. All snacking occasions occurring at different times during a 24 h record were then pooled to define the content and quality of overall daily snacking.

#### Computation of nutritional indicators of eating occasions

##### Energy intake and energy density

The total energy intake of each eating occasion was calculated by summing the energy intake of all food items ingested on that occasion. Energy density was defined as the ratio of energy intake by the weight of the eating occasion*100 [36]. Low-calorie beverages (first decile of energy per 100 g) were excluded from computation.

##### Nutrient density

To assess the nutrient density of eating occasions, the NRF9.3 index proposed by Fulgoni et al. [37] was used. The NRF9.3 is a score based on nine beneficial nutrients (protein, fibre, vitamins A, C and E) and minerals (magnesium, potassium, iron and calcium), and three nutrients that should be limited (saturated fat, added sugars and sodium). For each eating occasion, the total amount of each considered nutrient per 100 kcal was calculated using a published nutrient database [35] (without inclusion of dietary supplement intake); daily values defined by the Food and Drug Administration [38] were used to score each eating occasion using the NRF9.3 algorithm. A high positive score reflects dietary intake that provides large amounts of beneficial nutrients.

$$ {\displaystyle \begin{array}{l}\mathrm{Algorithm}:\mathrm{NRF}9{.3}_{100\ \mathrm{kcal}}={\sum}_{\mathrm{i}=1-9}\left({\mathrm{Nutrient}}_{\mathrm{i}}/{\mathrm{RDV}}_{\mathrm{i}}\right)\ast 100-{\sum}_{\mathrm{i}=1-3}\left({\mathrm{Nutrient}}_{\mathrm{i}}/{\mathrm{MDV}}_{\mathrm{i}}\right)\ast 100\\ {}\kern10.5em \mathrm{RDV}=\mathrm{recommended}\ \mathrm{daily}\ \mathrm{values};\mathrm{MDV}:\mathrm{maximum}\ \mathrm{daily}\ \mathrm{values}\end{array}} $$

### Contribution of food groups to total energy intake

We also computed the contribution of the food groups to energy intake by summing up the energy intake of each food item. Contribution in percent of food groups to energy intake on an eating occasion was assessed by calculating the amount of energy from each food group and dividing by the total energy intake of the eating occasion.

### Statistical analysis

Comparisons of demographic and socioeconomic characteristics between excluded and included participants were performed using chi-square or Fischer tests as appropriate. Data were weighted according to the French population socio-demographic distribution. Weighting was calculated separately for each sex using an iterative proportional fitting procedure and national census data on age, educational level, employment status, marital status, area of residence and presence of any children in the household [39]. Because of variable and unusual eating behaviors on the weekend, we focused our analysis on weekdays.

We used weighted percentages of individuals to describe occurrence of eating occasions and weighted means or medians for nutritional indicators to describe their nutritional quality.

Comparisons of energy intake, energy and nutrient densities between main meals, between snacks and between overall snack and main meals were performed using analysis of variance. Data pre-treatment and statistical analyses were performed using SAS (version 9.3; SAS Institute, Inc., Cary, NC, USA).

## Results

Among 144,746 individuals with available dietary data at baseline, we excluded individuals who were pregnant and those who did not provide at least two 24-h dietary records (*n* = 19,987; 13.8%), underreporting subjects (*n* = 15,785; 10.9%) and individuals with missing data for the weighting procedure (*n* = 4709; 3.3%), leaving 104,265 individuals for analysis. Among 104,265 individuals, after weighting, 47.6% were men and 52.4% were women; 20.6% of participants were ≤30 years and 26.4% were > 60 years; 27.7% had a primary school education level, while 13.0% had a postgraduate level (Table 1). Proportions of young subjects (18–30 years), individuals with primary school level, manual workers, employees and never employed persons, those belonging to the lowest income class were higher in excluded participants than those included in the sample while proportions of older subjects, those with postgraduate education, managerial staff individuals, and those belonging to the highest income class were lower (date not shown).

**Table 1:** Demographic and socioeconomic characteristics of the sample (weighted data) (*n* = 104,265)

	Weighted data (%)
Sex	
Men	47.6
Women	52.4
Age class	
18–30 y	20.6
31–45 y	27.9
46–60 y	25.1
< 60 y	26.4
Educational level	
Primary	27.7
Secondary	47.4
Undergraduate	11.9
Postgraduate	13.0
Occupational categories ^b^	
Never employed	3.2
Self-employed (craftsman, shopkeeper, company manager, farmer)	7.5
Managerial staff	19.6
Employees	32.4
Manual workers	15.2
Intermediate professions	22.1
Household income per consumption unit	
Unwilling to declare	11.8
< 1200 euros	24.0
1200–1800 euros	292
1800–2700 euros	20.2
> 2700 euros	14.8
Living area	
Rural	24.7
20,000 inhabitants.	16.6
20,000–200,000 inhabitants.	18.6
> 200,000 inhabitants	40.1
Household composition	
Living alone	16.2
Living with other adult(s) but no child	47.2
Living with at least one child	36.6

^a^Weighting accounted for socio-demographic characteristics compared with the national population census (age, occupational category, household including at least one child or not and living area)

^b^Unemployed and retired individuals were asked to indicate their last occupation status. In this particular case, the previous status was used to assess the occupation in the analyses

### Snacking occasions of weekdays

On weekdays, around 28% of participants snacked in the morning, 8% at midday, 45% during the afternoon, 18% in the evening and 10% at night. The highest values for energy intake from snacks and the lowest scores for nutrient density were found for afternoon, evening and late-night snacks while the morning snack showed the lowest mean energy load, the lowest mean energy density and the highest nutrient density score (Table 2). Hot beverages contributed most to the energy load of the morning snack (33%), followed by fatty-sweet and sugary products (13.6% and 15%) and fruits (8%) (Table 3). Fatty-sweet products contributed most to the energy intake of afternoon snacks (33%), followed by fruits (16%), hot beverages (11.4%) and sweet foods (9%) (Table 3). The main contributors to the energy content of the evening snack were fatty-sweet products (26%), fruits (11%), alcoholic beverages (9%) and sweet foods (8%) (Table 3). The highest contribution of sweetened drinks to energy intake from beverages was observed in the afternoon snack (34%) and the highest contribution of alcoholic beverages to energy intake from beverages was observed for the evening snack (27%) (Table 4).

**Table 2:** Nutritional characterization of meals and snacks of weekdays (*N* = 104,265)

Eating occasion	Subjects reporting the eating occasion (%)	Mean energy (SD) kcal	Median NRF9.3 score (SD)	Mean energy density without low-calorie beverages^a^ (kcal/100 g) (SD)
Meals				
Breakfast	86.0	414.0^b, d^ (233.8)	25.8^b, d^ (36.9)	175.9^b, d^ (99.6)
Lunch	95.6	734.6^b, d^ (350.6)	30.0^b, d^ (30.4)	137.9^b, d^ (52.5)
Dinner	96.3	734.0^b, d^ (390.3)	25.9^b, d^ (29.4)	133.9^b, d^ (57.3)
Snacks				
Overall	68.0	260.8^d^ (284.6)	22.8 (278.3) ^d^	222.2 (163.3) ^d^
Morning	27.8	104.1^c^ (155.0)	60.1 ^c^ (385.1)	211.3 ^c^ (170.2)
Midday	8.3	117.9 ^c^ (173.1)	53.1^c^ (328.8)	227.7 ^c^ (159.7)
Afternoon	44.5	192.4 ^c^ (203.5)	16.36^c^ (269.3)	223.9 ^c^ (156.3)
Evening	17.8	207.6^c^ (231.3)	12.8^c^ (595.2)	220.1 ^c^ (164.0)
Night	9.8	161.8^c^ (226.3)	11.1^c^ (1218.5)	223.9 ^c^ (179.1)

^a^10% lowest caloric beverages

^b^*P*-value < 0.001 (comparisons between main meals using analysis of variance)

^c^*P*-value < 0.001 (comparisons between snacks using analysis of variance)

^d^*P*-value < 0.001 (comparisons between overall snack and main meals using analysis of variance)

**Table 3:** Contribution (%) of food groups to energy intake of weekday snacking occasions

	Morning % (SD)	Midday % (SD)	Afternoon % (SD)	Evening % (SD)	Night time % (SD)	Overall % (SD)
Fruit	8.5 (26.5)	11.7 (27.7)	15.9 (32.0)	11.1 (26.9)	11.0 (29.5)	13.1 (28.0)
Bread	4.7 (16.6)	3.8 (14.1)	5.6 (16.8)	5.4 (16.0)	2.9 (12.6)	6.0 (16.4)
Milk and milk substitutes	7.2 (22.0)	2.1 (11.3)	2.7 (12.2)	2.1 (11.6)	4.8 (19.2)	3.9 (14.7)
Fatty-sweet products (pastry, cookies, chocolate, etc.)	13.6 (31.8)	15.2 (30.9)	33.3 (41.1)	25.9 (38.8)	27.3 (41.9)	29.6 (38.2)
Sweet foods (honey, candy, jam, etc.)	15.0 (32.0)	13.5 (29.1)	8.9 (23.8)	8.1 (23.7)	12.6 (30.5)	9.0 (22.5)
Hot beverages (coffee, tea, cappuccino, etc.)	32.9 (44.7)	24.7 (38.0)	11.4 (29.3)	5.9 (21.7)	7.1 (24.3)	11.3 (28.5)
Juices (fruit or vegetable)	5.3 (19.9)	4.3 (17.5)	2.3 (12.4)	3.1 (15.3)	4.0 (18.8)	3.5 (14.8)
Sweetened and light beverages (non- alcoholic)	3.4 (16.7)	5.1 (19.1)	4.6 (18.0)	5.0 (19.0)	7.1 (24.6)	4.4 (16.6)
Alcoholic beverages	0.1 (2.8)	2.6 (13.5)	1.9 (12.1)	9.2 (24.5)	6.0 (22.3)	3.2 (14.2)
Oleaginous seeds, appetizer	0.9 (8.7)	3.0 (13.9)	1.8 (11.1)	5.4 (18.4)	1.8 (12.1)	2.6 (12.6)

**Table 4:** Contribution of beverages to total energy intake of beverages (%) of weekday snacking occasions

	Morning snack % (SD)	Midday snack % (SD)	Afternoon snack % (SD)	Evening snack % (SD)	Night time snack % (SD)	Overall snack % (SD)
Milk and milk substitutes	22.9 (44.1)	7.3 (24.2)	13.3 (32.0)	8.2 (26.8)	18.8 (46.8)	13.0 (31.1)
Sweetened and light beverages (non-alcoholic)	6.0 (25.8)	26.9 (41.7)	34.2 (45.0)	29.8 (45.0)	26.4 (53.0)	13.1 (31.7)
Hot beverages (coffee, tea, cappuccino, etc.)	58.0 (52.9)	53.6 (46.7)	35.6 (45.4)	27.3 (43.8)	15.3 (41.2)	55.3 (47.3)
Juice (fruit or vegetable)	13.1 (35.5)	11.5 (30.1)	12.9 (31.6)	8.0 (26.7)	17.7 (44.7)	9.8 (27.7)
Alcoholic beverages	0.1 (3.3)	0.7 (7.7)	4.0 (18.8)	26.7 (43.5)	21.7 (50.3)	8.8 (26.8)

### Overall snack and main meals of weekdays

Overall, 68% of subjects snacked on at least one occasion while 86% of participants ate breakfast and around 96% ate lunch and 96% dinner during weekdays (Table 2).

Total energy intake from overall daily snacks (around 260 kcal) was lower than these from main meals (Table 2). Overall snack had a lower nutrient density score (22.8) than main meals (breakfast: 25.8 to lunch: 30.0); the mean energy density of overall daily snacks (222.2 kcal/100 g) was higher than main meals (dinner: 133.9 (57.3) to breakfast: 175.9 (99.6) kcal/100 g).

Mean energy intake at breakfast was around 414 kcal, and 734 kcal for both lunch and dinner. Median nutrient density score was higher for lunch compared to breakfast and dinner, while energy density was higher for breakfast than for lunch and dinner (Table 2).

Overall, fatty-sweet products (30%), fruits (13.1%), hot beverages (11.3%), sweet foods (9%) and bread (6%) contributed most to the energy intake from daily weekday snacks (Table 3). Hot or sweetened beverages and milk were the main contributors to the total energy intake of beverages for overall snack (Table 4).

Regarding breakfast on weekdays, food groups that contributed most to energy intake were bread, fatty-sweet products, sweet foods, butter, milk and breakfast cereals (Additional file 1: Table S1). The major contributors to total energy intake from beverages were hot beverages, milk and juices (Additional file 2: Table S2). For lunch and dinner on weekdays, fish, meat, poultry and eggs contributed most to the energy intake of for these meals (24 and 18%) (Additional file 1: Table S1). The other main food groups were starches and bread, followed by vegetables, fruits, cheese, sauces, fats and fatty-sweet products (Additional file 1: Table S1). During lunch and dinner, hot beverages, sweetened and “diet” drinks and alcoholic beverages were the main sources of energy intake from beverages. (Additional file 2: Table S2).

## Discussion

Our study showed that almost all adults ate the traditional three main meals (breakfast, lunch and dinner) on weekdays, while more than two-thirds of them also snacked at least once during the day, especially in the afternoon and, to a lesser extent, in the morning. Overall snack had lower nutrient density but higher energy density than main meals on weekdays. Energy density, nutrient density and food groups eaten strongly varied according to snack time. The morning snack represented the snacking occasion with the lowest energy density and the highest nutrient density, with high intake of hot beverages, fatty-sweet products and sugary foods while afternoon and evening snacks had the highest energy load, with high intake of fatty-sweet products and fruits. In addition, sweetened beverages strongly contributed to energy intake from beverages in afternoon and evening snacks. Alcoholic beverages were also an important contributor to energy intake from beverages in the evening snack.

Our findings confirm that snacking is a very frequent behavior among French adults during the week; indeed, 68% snacked at least once a day. This high percentage of individuals who snacked attained levels observed in previous work in the US [3]. Contrary to other countries, the French daily eating pattern is fairly synchronized [40], with three main meals per day [8], generally at set times and based on social eating habits [41]. Compared to a French representative survey, percentages of individuals eating a morning or an afternoon snack were higher in our study (28% and 45% vs. 10% and 35%) [42]. Such differences could be explained by important variations in data collection methods. The earlier study was based on a telephone interview and pre-defined questions. Moreover, some inherent biases in studies based on interviews, such as social desirability may be different in a web-based study. In particular, judgement bias due to higher perceived anonymity is probably lower in web-based studies and may encourage individuals to report more food items [33].

Contributions of fruits, sweetened beverages and fatty-sweet products to energy intake for overall snacking were higher in our study than in previous American and Finnish studies in adults, whereas the contribution of dairy products and bread was lower [18, 21]. In agreement with previous studies in adults in Finland and the United States [22, 43], our findings highlighted that energy intake and nutrient density are lower for most snacking occasions than for main meals, while energy density is higher. These results suggest that snacking is associated with less healthy choices. In addition, the fact that overall snack represented around 13% of total daily energy intake shows the importance of a better characterization of snacking occasions. The main food groups contributing to energy intake varied greatly between main meals and snacks. The major contribution of fatty-sweet products and sweet foods to the energy intake of snacking leads to intake of added sugars, lipids, saturated fatty acids and sodium, explaining the higher energy density and lower nutrient density of snacking compared to main meals. This important contribution of foods rich in nutrients that should be limited, along with high energy density during snacking**,** raise concern about the effect of snacking on weight and health status [44–48]. Mixed results, however, have been observed for associations between snacking and body mass index [12, 17, 19, 43, 49]. Further prospective analyses could help to define whether snacking involving high energy density is a risk factor of weight gain.

Snacking occasions had lower nutrient density (< 17) than main meals, except for morning and midday snacks. Evening and afternoon snacks only weakly contributed to the intake of recommended nutrients (fibre, vitamins, proteins and minerals), but provided nutrients the consumption of which should be limited based on dietary recommendations, such as saturated fatty acids, added sugars and sodium. This finding is related to the foods consumed on these eating occasions: fatty-sweet products, but also sweet foods and alcoholic beverages. Since percentages of individuals snacking in the evening and afternoon were relatively high in our sample, the associations between such behaviors and health events should be investigated to assess potential adverse effects. In contrast, fruits strongly contribute to the energy intake of snacks and may be beneficial [50]. Targeting both snacking and meal behaviors in public health messages is thus important.

The finding that most participants reported eating the main meals in a French population is concordant with previous studies conducted in French adults, which showed that the three-meal pattern continues to prevail among adults [9, 42, 51]. In our study, lunch was the main meal with the highest nutrient density. The higher contribution of cheese, alcoholic beverages and fast foods explains the lower nutrient density of dinner compared with lunch. As expected, food groups that contributed most to energy intake during lunch and dinner are animal foods (fish, meat, poultry and eggs) and cereals (starches and bread), followed by vegetables, fruits, cheese, sauces/added fats and fatty-sweet products. Our findings are concordant with previous studies regarding fish, meat and starches [5, 18], but differ in the contributions of fruits, vegetables and bread, which were higher in our population, while the contribution of milk was lower [18]. In France, main meals are generally structured into two or three courses, which could explain the wider variety of foods during a meal [42].

Since intakes of sugar-sweetened products, sugary drinks during snack eating occasions but also those of beneficial products such as fruits, appropriate actions are needed to promote consumption of healthy foods and limit other non-recommended items [1, 52]. Since snacking occasions are common, promoting intakes consumption of certain foods such as fruit when snacking, rather than banning snacks might be worth exploring. Since snacking could help to reduce hunger and improve satiety, favoring healthy snacks could potentially help to avoid overconsumption or to reduce energy intake during the subsequent meal, balancing daily intakes [12, 53–57]. In addition, a separate analysis showed that snacking behavior varies across demographic and socioeconomic subgroups. Although snacking was less prevalent in low socioeconomic categories and young adults, their snacks had higher energy content and were of poorer nutrient density. When focusing on public strategies regarding snacking behavior, policy makers should take into account these demographic and socio-economic disparities to implement specific actions – either through professional networks or education programs for instance.

### Strengths and limitations

A major strength of our study lies in its assessment of eating habits of French adults, taking into account both meals and snacks. Characterizing eating occasions on their daily timing allowed us to describe snacks more precisely. Further analyses are however needed to assess potential correlations between multiple snacking occasions within subjects that may have affected the variability of our results. The different parameters used to assess the nutritional content of such eating occasions (contribution of different food groups to snacking energy, nutrient and energy densities) constitute an original contribution; indeed, previous works had mainly focused on content of specific nutrients and energy. Since the NRF9.3_100kcal_ has been validated and can be applied to individual food, meals, menus and even the daily diet, this nutrient index enabled us to assess the nutrient density of the different eating occasions [37, 58]. The strength of this score lies in the fact that it includes both positive and negative components. Since the NutriNet-Santé cohort includes volunteers, subjects had a healthier lifestyle and were probably more interested in nutrition than the general population. Thus, caution is needed when interpreting and generalizing results. Analyses, however, were weighted according to French population socio-demographic distribution, which allows bias to be limited. A web-based design may affect internal validity by inducing misreporting. However, studies investigating the validity of our web-based, self-reported dietary record tool against biomarkers showed that our tool performs well at estimating several nutrients and food intakes [31, 32]. Because of the lack of a consensual definition of snacking, our definition based on time of day, along with our analytical choices, is subject to caution. Because of variable and unusual eating behaviors on weekends, we chose to relate findings of weekdays only. Further analyses describing and comparing all eating occasions on weekends could usefully complement our current results. Contrary to previous work, the definition of snacking was not based on consumed food items or the participant’s definition of snacking. In addition, the fact that the volunteers did not declare snacking occasions as such (but only eating occasions in general) may have reduced the desirability bias, since snacking is often viewed as unhealthy behavior.

## Conclusions

This study highlights the common practice of snacking among French adults, but indicates that most individuals still eats the three main meals. Snack content varies greatly according to timing. The morning snack appears to be heathier than afternoon and evening snacks. The high prevalence of energy-dense, low-nutrient snacks raises concern, as it may be a risk factor in weight gain [47]. Snacking was associated with intake of foods and nutrients whose consumption should be limited, but also foods recommended for a healthy diet, such as fruits. Better knowledge of the effect of snacking behavior on weight and health status is crucial for defining public action in promoting healthy eating habits.

## Additional files

Additional file 1: **Table S1.** Contribution of food groups to energy intake of main meals of weekdays. (DOCX 15 kb)

Additional file 2: **Table S2.** Contribution of beverages to total beverage consumption at main meals of weekdays. (DOCX 14 kb)

## Abbreviations

MDV: Maximum daily values
NRF9.3 _100kcal_: Nutrient-rich food index 9.3 per 100 kcal
RDV: Recommended daily values
