# Supplementary material for: Energy, nutrient and food content of snacks in French adults
Source: Nutr J. 2018 Feb 27;17:33. doi: 10.1186/s12937-018-0336-z (PMC5828417; doi:10.1186/s12937-018-0336-z)
Supplement: Supplementary file 1 — Table S1. Contribution of food groups to energy intake of main meals of weekdays. (DOCX 15 kb) [file 12937_2018_336_MOESM2_ESM.docx]

**Additional file 2: Table S2:** Contribution of beverages to total beverage consumption at main meals of weekdays

|  | Breakfast  % (SD) | | Lunch  % (SD) | Dinner  % (SD) |
| --- | --- | --- | --- | --- |
|  | Contribution of beverage group to total energy intake of beverages | | | |
| Milk and milk substitutes | 28.4 (40.5) | 4.3 (21.4) | | 3.4 (19.6) |
| Sweetened and light beverages (non-alcoholic) | 5.3 (20.2) | 24.7 (46.5) | | 26.5 (48.2) |
| Water and hot beverages (coffee, tea, cappuccino, etc.) | 46.8 (46.6) | 35.9 (51.6) | | 12.7 (36.6) |
| Juice (fruit or vegetable) | 19.3 (35.1) | 5.6 (24.8) | | 4.8 (23.3) |
| Alcoholic beverages | 0.2 (3.7) | 29.5 (49.3) | | 52.6 (54.6) |
