# Supplementary material for: Energy, nutrient and food content of snacks in French adults
Source: Nutr J. 2018 Feb 27;17:33. doi: 10.1186/s12937-018-0336-z (PMC5828417; doi:10.1186/s12937-018-0336-z)
Supplement: Supplementary file 2 — Table S2. Contribution of beverages to total beverage consumption at main meals of weekdays. (DOCX 14 kb) [file 12937_2018_336_MOESM1_ESM.docx]

Additional file 1: Table S1: Contribution of food groups to energy intake of main meals of weekdays

|  | Contribution to energy intake % (SD) | | |
| --- | --- | --- | --- |
|  | Breakfast | Lunch | Dinner |
| Fruits | 4.8 (12.4) | 5.5 (9.8 ) | 5.2 (9.5) |
| Vegetables | 0.2 (2.42) | 7.6 (12.3 ) | 9.0 (14.3) |
| Starches (rice, pasta, etc.) | 0.3 (2.93) | 15.9 (19.2 ) | 12.1 (18.4) |
| Bread | 30.4 (25.05) | 10.4 (12.7 ) | 10.9 (13.0) |
| Fish, meat, poultry, eggs | 1.1 (6.60) | 24.1 (20.0 ) | 18.1 (20.0) |
| Milk and milk substitutes | 7.0 (12.72) | 0.2 (2.1 ) | 0.3 (2.7) |
| Cheese | 1.5 (7.46) | 5.3 (9.5 ) | 7.0 (11.8) |
| Yogurts | 4.2 (12.30) | 2.9 (6.8 ) | 4.0 (8.8) |
| Dairy desserts | 1.1 (6.83) | 3.1 (9.1 ) | 3.2 (9.2) |
| Breakfast cereals | 6.5 (17.96) | 0.2 (3.1 ) | 0.3 (3.8) |
| Fatty-sweet products (pastry, cookies, chocolate, etc.) | 13.9 (27.82) | 5.6 (13.7 ) | 6.4 (15.7) |
| Sweet foods (honey, candy, jam, etc.) | 9.0 (12.08) | 1.2 (4.0 ) | 1.5 (5.0) |
| Hot beverages (coffee, tea, cappuccino, etc.) | 3.6 (10.06) | 0.2 (1.4 ) | 0.1 (1.7) |
| Juice (fruit or vegetable) | 4.3 (8.94) | 0.2 (2.1 ) | 0.2 (2.0) |
| Sweetened and light beverages (non-alcoholic) | 1.3 (5.61) | 0.8 (4.0 ) | 0.9 (4.0) |
| Alcoholic beverages | 0 (0.48) | 2.1 (5.8 ) | 4.1 (9.0) |
| Oleaginous seeds, appetizer | 1.0 (6.21) | 0.8 (5.0 ) | 1.7 (7.3) |
| Fast food (pizza, burgers, etc.) | 0.1 (2.43) | 3.8 (14.7 ) | 6.7 (19.2) |
| Butter and margarine | 8.7 (12.38) | 1.5 (4.4 ) | 1.4 (4.3) |
| Oil, sauces and other fats | 0.2 (2.19) | 5.9 (9.5 ) | 5.9 (9.9) |
